# RNA sequencing analysis of activated macrophages treated with the anti-HIV ABX464 in intestinal inflammation

**DOI:** 10.1038/sdata.2017.150

**Published:** 2017-10-17

**Authors:** Laurent Manchon, Karim Chebli, Laura Papon, Conception Paul, Aude Garcel, Noëlie Campos, Didier Scherrer, Hartmut Ehrlich, Michael Hahne, Jamal Tazi

**Affiliations:** 1 IGMM, CNRS, Univ. Montpellier, 34293 Montpellier, Cedex 5, France; 2 ABIVAX, 1919 route de Mende, Montpellier, Cedex 5 34293, France

**Keywords:** High-throughput screening, Data processing, Data acquisition

## Abstract

RNA-Seq enables the generation of extensive transcriptome information providing the capability to characterize transcripts (including alternative isoforms and polymorphism), to quantify expression and to identify differential regulation in a single experiment. To reveal the capacity of new anti-HIV ABX464 candidate in modulating the expression of genes, datasets were generated and validated using RNA-seq approach. This comprehensive dataset will be useful to deepen the comprehensive understanding of the progression of human immunodeficiency virus (HIV) associated with mucosal damage in the gastrointestinal (GI) tract and subsequent inflammation, providing an opportunity to generate new therapies, diagnoses, and preventive strategies.

## Background & Summary

RNA sequencing (RNA-seq) transcriptome analysis using massively parallel next generation sequencing technology provides the capability to understand global changes in gene expression throughout a range of tissue samples and have become the gold standard for in depth characterization of novel transcript isoforms. RNA-seq (Quantification) is used to analyse gene expression of certain biological objects under specific conditions. It is a cost-effective quantification method that produces high reproducibility, high accuracy and wide dynamic range. As described in our related manuscript submitted earlier in Scientific Reports^1^, our aim in this study is to consolidate the protective effect of ABX464 in DSS-induced colitis comparing transcript levels between bone marrow-derived macrophages (BMDMs) ABX464-treated stimulated or not by LPS. To perform this task we sequenced here 24 samples of C57BL6 strains of mice using BGI RNA sequencing (RNA-seq) technology^2,3^.

ABX464 is a first-in-class antiviral drug candidate used to treat patients infected with human immunodeficiency virus. ABX464 is an orally available, well-tolerated small molecule that blocks HIV replication through the modulation of RNA biogenesis. ABX464 demonstrates anti-viral activity in treatment-naïve patients and induces a long-lasting control of the viral load in HIV-infected humanized mice after treatment arrest. This treatment might improve current therapies by hampering the appearance of virus resistance and, more importantly, lead to a long-lasting control of HIV.

Despite the successful control of viremia, many HIV-infected individuals treated with antiretroviral therapy (ART) exhibit residual inflammation associated with non-AIDS-related morbidity and mortality. Several reports have shown that measures of inflammation and immune activation are the best independent predictors of disease progression in HIV-infected individuals. It is also known that elevated inflammation and immune activation predict non-AIDS-associated morbidities and mortality, even in well-suppressed treated HIV-infected individuals. Mucosal damage to the gastrointestinal (GI) tract with resulting microbial translocation significantly contributes to the heightened and persistent chronic inflammation and immune activation characteristic to HIV infection and might contribute to virus persistence during ART. Recently, a dextran sulfate sodium (DSS)-induced nonhuman primate (NHP) colitis model in SIV-infected rhesus macaques a natural host species for SIV that does not manifest GI tract damage or chronic immune activation during infection, was used to demonstrate that DSS-exposure results in colitis with elevated levels of plasma SIV RNA, sCD14, LPS, CRP and mucosal CD4 T cell loss^4,5^. The pathogenic potential of bacterial translocation has also been studied in humanized mice exposed to DSS to provide evidence that HIV chronic infection contributes to systemic inflammation under DSS-exposure^6,7^. Given the similarities in colitis induction between DSS-exposed macaques and mice^5^, the antiviral activity of ABX464 in humanized mice model^8^ and its pharmacodynamics and pharmacoketics profiles in rodent, NHP^8^ and humans^9^, the anti-inflammatory properties of this drug in DSS-treated mice were tested (Chebli *et al.*). The results presented in this manuscript based on the use of DSS-induced colitis in mice, show for the first time that an anti-HIV drug can specifically act on the immune system to resolve the inflammation.

The principal aim of this study was to provide a comprehensive resource to deposit the raw RNA-seq data sets underpinning the advanced high-resolution transcriptome analysis of ABX464-treated BMDMs stimulated with LPS. To this end the data from three distinct group of BMDM cultures were analyzed for different gene expression levels using RNA-seq. We performed a high-resolution transcriptome analysis of ABX464-treated BMDMs stimulated or unstimulated with LPS. In this work, the RNA-seq libraries were sequenced using the BGISEQ-500 platform which use the DNA nanoball technology. Approximately 1 billion 50-bp single-end RNA-seq reads were generated, with an average of over 45 million sequence reads per sample.

24 intestinal macrophages mRNA profiles of three distinct group of BMDM cultures from mice were generated by deep sequencing, using Illumina protocol. The sequence reads that passed quality filters were analyzed at the transcript isoform level with two methods: HISAT^10^ and Bowtie2 (ref. 11) followed by genes expression quantification using a software package called RSEM^12^.

Using an optimized data analysis workflow, about 45 million sequence reads per sample were mapped to the mouse genome (build mm9) and identified 1,383 expressed genes in the LPS-stimulated BMDMs among which 439 and 397 genes increased and decreased in expression >=1.5 log2-fold, respectively. Altered expression of some genes was confirmed with qPCR, demonstrating the high degree of sensitivity of the RNA-seq method. Hierarchical clustering of differentially expressed genes uncovered several as yet uncharacterized genes that may contribute to inflammatory function. Data analysis with HISAT^10^/Bowtie2 (ref. 11) and RSEM^12^ workflows revealed a significant overlap yet provided complementary insights in transcriptome profiling. A comprehensive RNA-seq data resource was provided on the anti-inflammatory properties of ABX464 that warrant exploration in both HIV and inflammatory ulcerative colitis (UC) disease. The data generated here will be valuable to the vision research community for characterizing global changes in gene expression and facilitate a better understanding of the complexities during anti-AIDS drugs treatment and then can help to develop better ways to combat HIV.

## Methods

### Macrophages RNA-seq preparation

Cell isolates from the femurs of C57BL6 mice (in Table 1 column sample; 58, 67, 69 stands for the number of the mouse from which cells were derived, the final number 1–8 stands for the different stimulation time points and treatments) were cultured for 9 days in the presence of GM-CSF (50 ng ml^−1^; Peprotech). Cell cultures were treated for the last 3 days with either ABX464 (5 μM) suspended in DMSO or DMSO alone. After additional stimulation for 6 h with LPS (4 μg ml^−1^; Invivogen) or unstimulation (NS), the supernatants were analyzed for cytokine production using a cytometric bead array technology (CBA; Becton Dickinson). The detection limits were 5 pg ml^−1^ for IL-6, 52.7 pg ml^−1^ for MCP-1, 17.5 pg ml^−1^ for IL-10, 7.3 pg ml^−1^ for TNF α, and 10.7 pg ml^−1^ for IL12p70 (Results presented in ref. 1). The cells were lysed in the Qiazol and the RNAs were purified with the miRNeasy kit from Qiagen. Dosage and analysis are performed using Nanodrop/Qubit and 2,100 Bioanalyzer (Agilent Technologies).

### RNA-seq library preparation and sequencing

DNase I was used to digestion step, DNase I degrade double-stranded and single-stranded DNA contaminant in RNA samples. RNA molecules were purified using oligo (dT)-attached magnetic beads. The RNA intensity was checked using a 2,100 Bioanalyzer (Agilent Technologies) and only high quality samples with an RNA Integrity Number (RIN) value greater than or equal to 7 were used to construct the sequencing library. Then mRNA were fragmented into small pieces using fragmentation reagent. In cDNA synthesis first-strand cDNA was generated using random hexamer-primed reverse transcription, then was followed by a second-strand cDNA synthesis. Typically, 1 μg of total RNA was used with the TruSeq RNA library preparation kit (Illumina) in accordance with low-throughput protocol, except that SuperScript III reverse transcriptase (Invitrogen) was used to synthesize first strand cDNA. In the step of end repair the synthesized cDNA was subjected to end-repair and then was 3′ adenylated. Adaptors were ligated to the ends of these 3′ adenylated cDNA fragments. We used PCR to amplify the cDNA fragments with adaptors from previous step. PCR products are purified with the SPRI beads, and dissolved in EB solution. In the circularization step the double stranded PCR products were heat denatured and circularized by the splint oligo sequence. The single strand circle DNA (ssCir DNA) were formatted as the final library. After PCR enrichment and purification of adapter-ligated fragments, the concentration of DNA with adapters was determined with quantitative PCR (Applied Biosystems 7,500) using primers QP1 5′- AATGATACGGCGACCACCGA-3′ and QP2 5′- CAAGCAGAAGACGGCATACGAGA-3′. The length of the DNA fragment was measured using a 2,100 Bioanalyzer.

The library was amplified with phi29 to make DNA nanoball (DNB) which have more than 300 copies of one molecular. The DNBs were load into the patterned nanoarray and single end 50 bases reads were generated in the way of sequenced by synthesis. Then, RNA-seq libraries were sequenced using the BGISEQ-500 sequencer. Base-calling was performed by the BGISEQ-500 software version 0.3.8.1111.

### Data filtering

‘Dirty’ raw reads were defined as reads which contain the sequence of adaptor, high content of unknown bases and low quality reads. These reads were removed before downstream analysis to decrease data noise. Filtering steps are as follows:

The quality of the RNA-seq libraries was first evaluated using the FastQC v0.11.5 software. Then, the reads were subjected to standard quality control (QC) criteria according to the following parameters: (1) trimming and cleaning reads that aligned to primers and/or adaptors, (2) reads with over 50% of low-quality bases (quality value ≤5) in one read, and (3) reads with over 10% unknown bases (N bases). After filtering, the remaining reads are called ‘clean reads’ and stored as FASTQ format^13^.

### RNA-seq data analysis

24 samples of C57BL6 strains of mice were sequenced using RNA-Seq technology^2,3^, averagely generating 45,188,386 raw sequencing reads and then 45,120,825 clean reads after filtering low quality (see Data Filtering in method page). Table 1 briefly summarizes information of sequencing data for each sample.

After filtering, clean reads are mapped to reference using HISAT^10^/Bowtie2 (ref. 11) tools. The average mapping ratio with reference gene is 83.67% and Table 2 lists separate mapping rate for each sample. The average genome mapping ratio is 91.47%. Strict quality control was conducted for each sample to evaluate whether the sequencing data are qualified.

Sequencing data saturation analysis is used to measure whether the depth of sequencing data is sufficient for informatics analysis. With the number of sequenced reads increasing, the number of identified genes is also increased. However, when the number of sequenced reads reaches a certain amount, the growth curve of identified genes flattens, indicating that the number of identified genes tends to reach the saturation.

**Figure 1:**
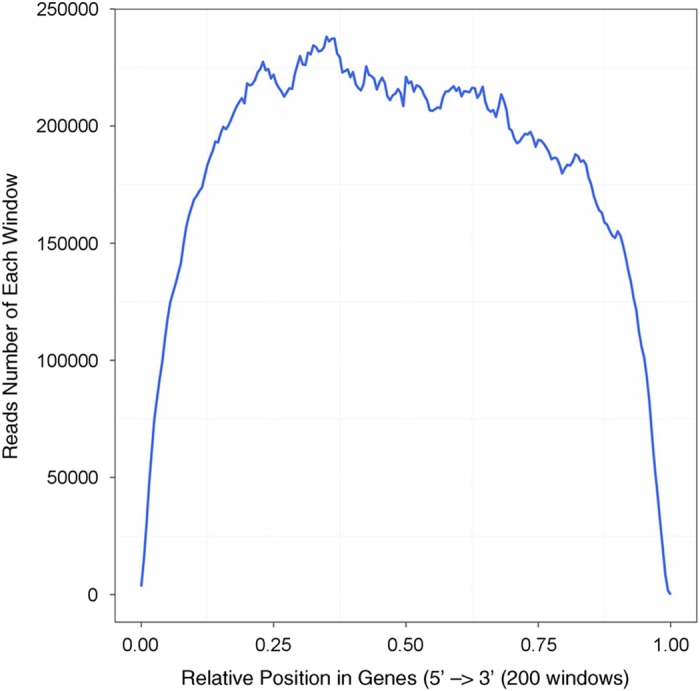
Reads distribution on reference gene. Because of variable lengths of reference genes, the average length of genes is divided into N equal parts. Each equal part is called a window. X-axis shows the relative position of genes, and Y-axis shows the number of reads in each window.

The distribution of the reads on reference gene reflects whether each part of the gene body is evenly sequenced. If the randomness is good, the reads in every position (from 5' terminal to 3' terminal) would be evenly distributed. If the randomness is poor, reads preference to specific gene region will directly affect subsequent bioinformatics analysis. We use the distribution of reads on the reference genes to evaluate the fragmentation randomness, as shown in Fig. 1.

**Figure 2:**
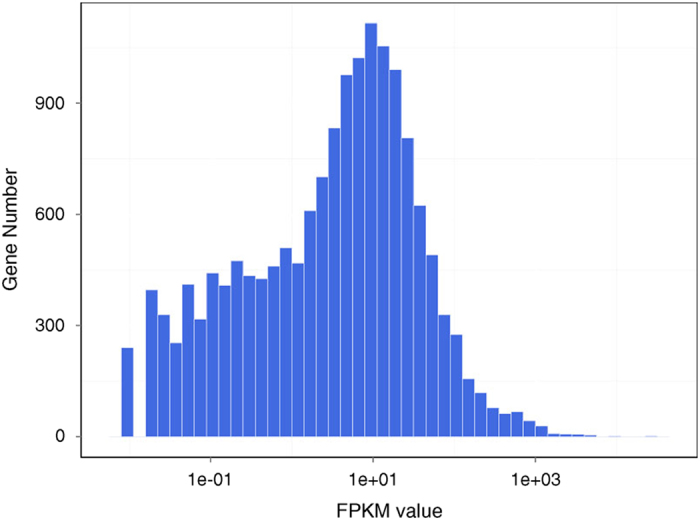
Histogram distribution of genes on expression level of each sample. X-axis is FPKM value (the coordinate has been changed by logarithm for better view). Y-axis is gene number of corresponding FPKM.

Gene’s expression level is quantified by a software package called RSEM^12^. The number of identified expressed genes were counted and its proportion to total gene number in database for each sample was calculated. Meanwhile, the distribution of gene number on different expression level for each sample are shown as Fig. 2, to get a general idea about the expression levels of each gene. The file all.gene.FPKM.xls available on Figshare repository is an expression table for all samples with brief gene description and annotation.

DEGs (Differentially Expressed Genes) screening is aimed to find differential expressed genes^14^ between samples and perform further function analysis on them. Poisson distribution method was used to perform this analysis.

All expressed genes of each pairwise are stored in *.GeneDiffExp.xls and screened DEGs are stored in *.GeneDiffExpFilter.xls. They have the same file format and are listed in *Figshare* repository (Data Citation 1).

**Figure 3:**
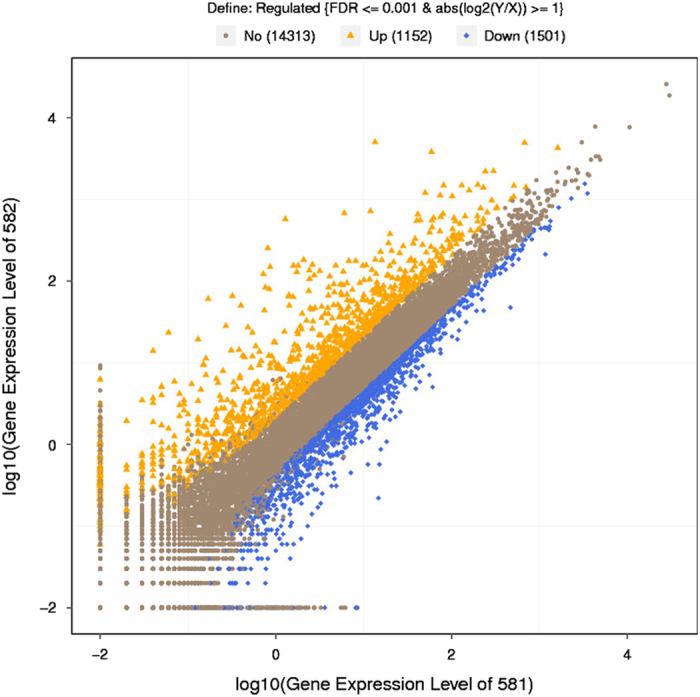
Scatter plots of all expressed genes in each pairwise. X-axis and Y-axis present log2 value of gene expression. Blue means down-regulation gene, orange means upregulation gene and brown means non-regulation gene. If a gene expressed just in one sample, its expression value in another sample will be replaced by the minimum value of all expressed genes in control and case samples. Screening threshold is on top legend.

**Figure 4:**
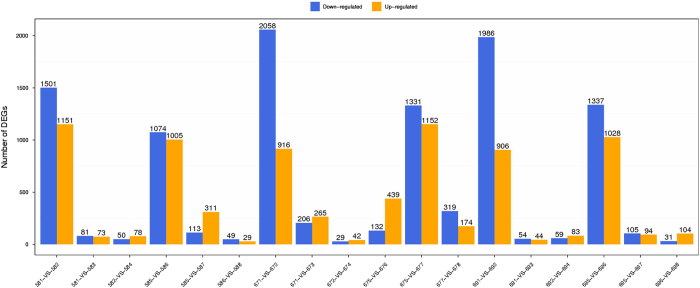
Statistic of differentially expressed genes. X axis represents pairwise and Y axis means number of screened DEGs. Blue bar denotes down-regulated genes and orange bar for the up-regulated.

For result list of each control-treatment pair above (*.GeneDiffExp.xls), scatter plots were draw for all expressed genes (Fig. 3) to present the distribution of DEGs in screening threshold dimensions. Also, a histogram is shown to demonstrate the number of genes that are significantly up-down in each pairwise comparison (Fig. 4).

**Figure 5:**
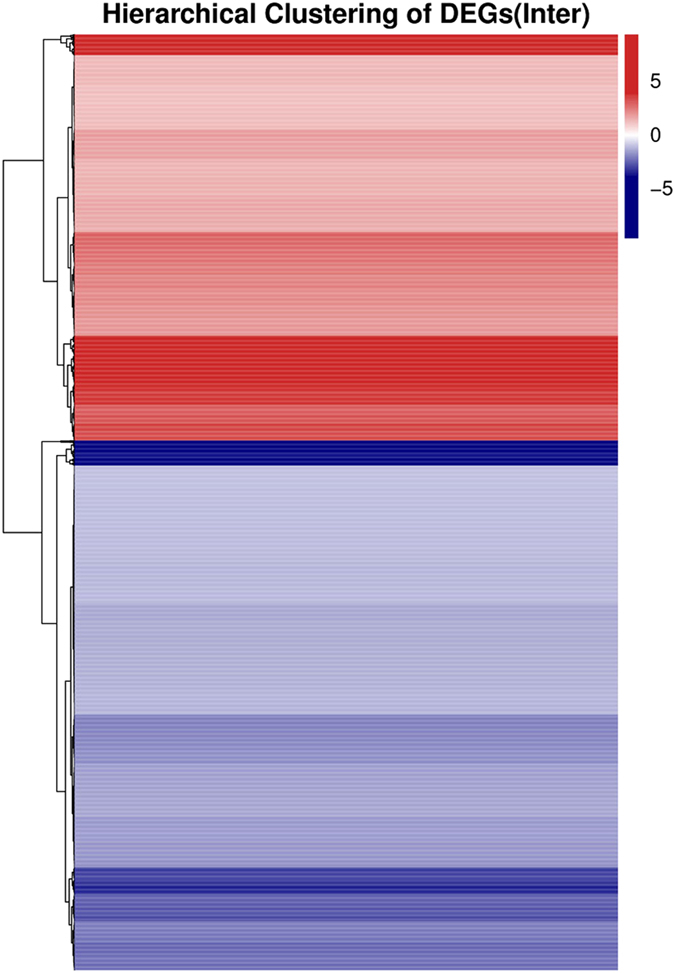
Intersection heatmap of DEGs for each cluster plan. Only genes that differentially expressed in all pairwises of cluster plan are used to build this heatmap. Gradient color barcode at the right top indicates log2 (FC) value (FC, Fold-Change of expression in treatment case to expression in control case). Each row represents a DEG and each column represents a condition pairwise (for some reason in R method, if there is only one pairwise, the pairwise name doesn’t appear in bottom). DEGs with similar fold-change value are clustered both at row and column level.

Genes with similar expression patterns usually have same functional correlation. We also provide clustering analysis of differentially expressed genes with cluster^15,16^ and javaTreeview software^17^ according to the provided cluster plans for DEGs. Complete intersection and union DEGs heatmap for each cluster plan were provided (Fig. 5).

Annotation analysis of Gene Ontology (GO) are performed for screened DEGs and then generate a report named *GOView.html* under the data folder of *Figshare* (Data Citation 1).

After getting GO annotation for DEGs, WEGO software^18^ was used to do GO functional classification to help understand the distribution of gene functions of the specie from the macro level.

**Figure 6:**
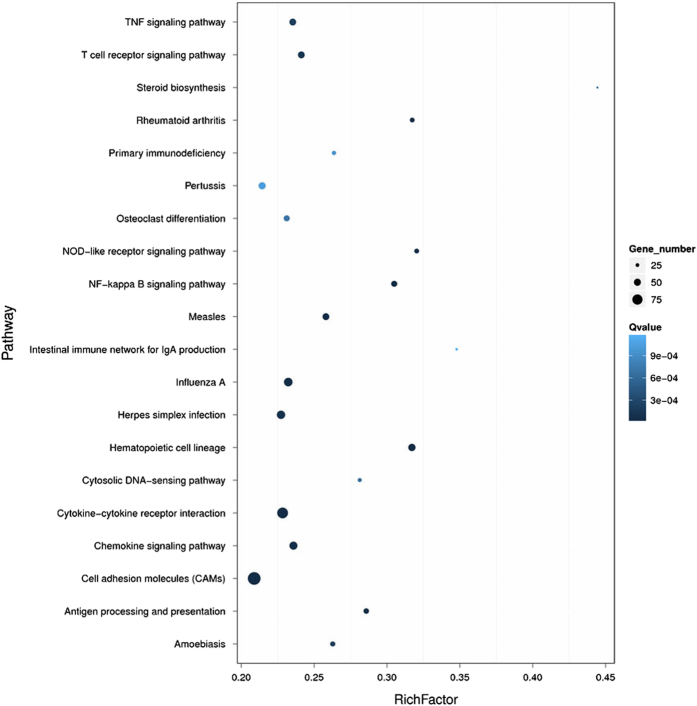
Statistics of pathway enrichment of DEGs in each pairwise. RichFactor is the ratio of differentially expressed gene numbers annoted in this pathway term to all gene numbers annoted in this pathway term. Greater richFator means greater intensiveness. Qvalue is corrected pvalue ranging from 0~1, and less Qvalue means greater intensiveness. We just display the top 20 of enriched pathway terms.

**Figure 7:**
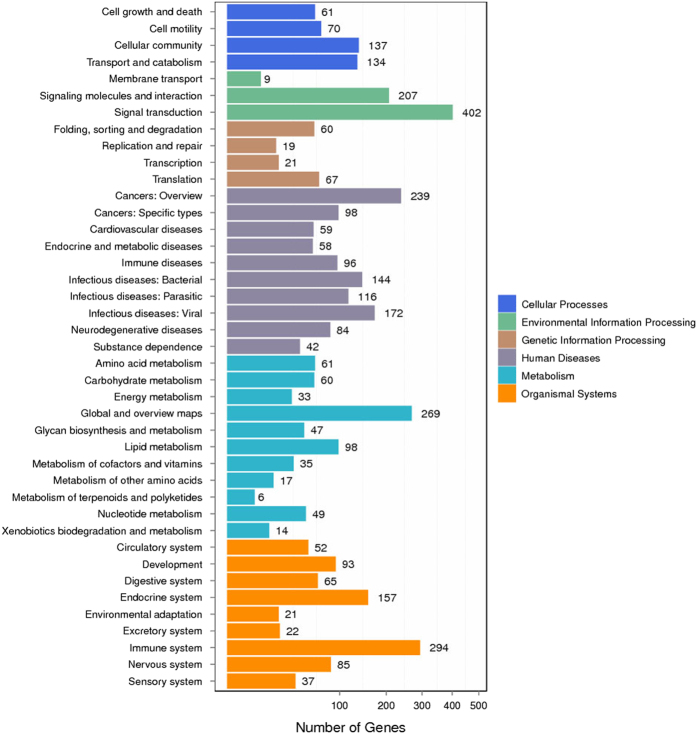
KEGG classification on DEGs for each pairwise. X axis means number of DEGs. Y axis represents second KEGG pathway terms. All second pathway terms are grouped in top pathway terms indicated in different color.

Genes usually interact with each other to play roles in certain biological functions. We perform pathway enrichment analysis of DEGs based on KEGG database^19^ and generate a report for DEGs in each pairwise respectively, stored in *Figshare* folder (Data Citation 1). In addition, we generate a scatter plot for the top 20 of KEGG enrichment results (Fig. 6) and a bar plot for the statistics of KEGG terms types (Fig. 7).

Different proteins often form complex proteins through complicated interactions to perform their biological functions. Protein-protein interaction network analysis integrates several famous interaction network databases such as BIND, BioGrid and HPRD and constructs interaction networks for DEGs' coding proteins. The analysis result documents are showed by Medusa software^20^ and webpage version is presented by the report *network_en.html* under *Figshare* folder (Data Citation 1).

### Statistical data analysis

Referring to ‘The significance of digital gene expression profiles’^14^, we have developed a strict algorithm to identify differentially expressed genes between two samples. Denote the number of unambiguous clean tags (which means reads in RNA-seq) from gene A as x, given every gene's expression occupies only a small part of the library, x yields to the Poisson distribution:

$$p(x)=\frac{e^{-\lambda }\lambda^{x}}{x!}\;(\lambda \;\mathrm{is}\;\mathrm{the}\;\mathrm{real}\;\mathrm{transcript}\;\mathrm{of}\;\mathrm{the}\;\mathrm{gene})$$

The total clean tag number of the sample 1 is N1, and total clean tag number of sample 2 is N2; gene A holds x tags in sample 1 and y tags in sample 2. The probability of gene A expressed equally between two samples can be calculated with:

$$\begin{array}{l} 2\sum_{i=0}^{i=y}p(i|x)\\ or\;2\times (1-\sum_{i=0}^{i=y}p(i|x))(if\sum_{i=0}^{i=y}p(i|x)>0.5)\\ p\left(y|x\right)={\left(\frac{N_{2}}{N_{1}}\right)}^{y}\frac{\left(x+y\right)!}{x!y!{\left(1+\frac{N_{2}}{N_{1}}\right)}^{\left(x|y|1\right)}} \end{array}$$

We do correction on *P*-value to differential gene expression test using Bonferroni method^21^. Since DEG analysis generate a large multiplicity problems in which thousands of hypothesis (is gene x differentially expressed between the two groups) are tested simultaneously, correction for false positive (type I errors) and false negative (type II) errors are performed using FDR method^22^. Assume that we have picked out R differentially expressed genes in which S genes really show differential expression and the other V genes are false positive. If we decide that the error ratio ‘Q=V/R’ must stay below a cutoff (e.g., 5%), the FDR was set to a number no larger than 0.05. We use 'FDR ≤0.001 and the absolute value of Log2Ratio ≥1' as the default threshold to judge the significance of gene expression difference. More stringent criteria with smaller FDR and bigger fold-change value can be used to identify DEGs.

### Code availability

FastQC v0.11.5 software.

Available online at: http://www.bioinformatics.babraham.ac.uk/projects/fastqc

## Data Records

The RNA-seq data from this study have been submitted and archived to the NCBI Gene Expression Omnibus (GEO; http://www.ncbi.nlm.nih.gov/geo/) under sample accession number GSE97062 (Data Citation 2).

In addition, the complete datasets, including expression profiles (Data Citation 1), figures and report analysis files (Data Citation 3, 4, 5) are available on the Figshare data management platform.

Data Citation 1 describe quantitative analysis with all the figures and tables relative to each gene. Data Citation 3 provide supplementary material about statistical analysis such as tables and graphs to show in detail up and down regulated genes in LPS-stimulated BMDMs. Data Citation 4 show in Excel tables the results (quality, saturation) of mapping step for each library. Finally, Data Citation 5 is dedicated to functions analysis to highlight expression, distribution of gene and their potential role in tissue.

## Technical Validation

### Quality control—RNA integrity

To determine the quality of the RNA-seq libraries, RNA intensity was checked using a 2,100 Bioanalyzer (Agilent Technologies). The integrity of the total RNA was calculated by the RNA Integrity Number (RIN) algorithm, which can be used to estimate RNA quality and degradation level. Higher RIN values indicate better integrity of total RNA, with the highest RIN value being 10. In this study, only total RNAs with a RIN value of greater than or equal to 7 were used to construct the sequencing library, which shows RNA with high integrity was used.

### Quality validation and analyses

We applied FastQC v0.11.5 software to determine data quality and analysed several measures, including the sequence quality per base, GC content per sequence, sequence duplication levels, and quality score distribution over all sequences of the fastq files^13^. A representative summary plots are depicted in Figshare repository (Data Citation 3). Here, the quality scores per base were high, with a median quality score above 30 suggesting high quality sequences across all bases. The GC distribution per base over all sequences was examined. The GC composition pattern was similar to theoretical distribution indicating that the samples were contaminant free. In addition, sequence complexity was examined. Approximately 15% of sequences were shown over 10 to 100 times, either from highly expressed transcripts duplications. The quality score distribution over all sequences was analysed to see if a subset of sequences had universally poor quality. The average quality for most sequences was high, with scores above 37, which indicated that a significant proportion of the sequences in a run had overall high. All other fastqc files were shown to have similar quality metrics.

## Additional Information

**How to cite this article:** Manchon, L. *et al.* RNA sequencing analysis of activated macrophages treated with the anti-HIV ABX464 in intestinal inflammation. *Sci. Data* 4:170150 doi: 10.1038/sdata.2017.150 (2017).

**Publisher’s note:** Springer Nature remains neutral with regard to jurisdictional claims in published maps and institutional affiliations.

## Supplementary Material



## Figures and Tables

**Table 1:** Summary of sequencing data for each sample.

**Sample**	**Sequencing Strategy**	**Time Point**	**Stimulation**	**Treatment**	**Raw Data Size (bp)**	**Raw Reads Number**	**Clean Data Size (bp)**	**Clean Reads Number**	**Clean Data Rate (%)**
581	SE50	12 h	NS	DMSO	2177780600	43555612	2177704450	43554089	99.99
582	SE50	12 h	+ LPS	DMSO	2286641850	45732837	2284394800	45687896	99.90
583	SE50	12 h	NS	ABX464	2286729500	45734590	2286667900	45733358	99.99
584	SE50	12 h	+ LPS	ABX464	2286677050	45733541	2272614700	45452294	99.38
585	SE50	48 h	NS	DMSO	2177833800	43556676	2177769250	43555385	99.99
586	SE50	48 h	+ LPS	DMSO	2286674400	45733488	2271449550	45428991	99.33
587	SE50	48 h	NS	ABX464	2286672950	45733459	2278218550	45564371	99.63
588	SE50	48 h	+ LPS	ABX464	2286705150	45734103	2284648050	45692961	99.91
671	SE50	12 h	NS	DMSO	2286656900	45733138	2277603650	45552073	99.60
672	SE50	12 h	+ LPS	DMSO	2177390000	43547800	2177332500	43546650	99.99
673	SE50	12 h	NS	ABX464	2286644550	45732891	2286555900	45731118	99.99
674	SE50	12 h	+ LPS	ABX464	2286680700	45733614	2286621650	45732433	99.99
675	SE50	48 h	NS	DMSO	2286664900	45733298	2286609750	45732195	99.99
676	SE50	48 h	+ LPS	DMSO	2286695750	45733915	2285342400	45706848	99.94
677	SE50	48 h	NS	ABX464	2286657850	45733157	2285755750	45715115	99.96
678	SE50	48 h	+ LPS	ABX464	2177678700	43553574	2177622700	43552454	99.99
691	SE50	12 h	NS	DMSO	2286665700	45733314	2278600650	45572013	99.64
692	SE50	12 h	+ LPS	DMSO	2177614000	43552280	2177556650	43551133	99.99
693	SE50	12 h	NS	ABX464	2286655650	45733113	2284221650	45684433	99.89
694	SE50	12 h	+ LPS	ABX464	2286686550	45733731	2286455400	45729108	99.98
695	SE50	48 h	NS	DMSO	2177711450	43554229	2177646300	43552926	99.99
696	SE50	48 h	+ LPS	DMSO	2286707650	45734153	2279763650	45595273	99.69
697	SE50	48 h	NS	ABX464	2286548450	45730969	2286495900	45729918	99.99
698	SE50	48 h	+ LPS	ABX464	2286689250	45733785	2277338850	45546777	99.59
Clean Data Rate (%)=Clean Reads Number/Raw Reads Number.									

**Table 2:** Alignment statistics of reads align to reference gene.

**Sample**	**Total Reads**	**Total Mapped Reads (%)**	**Unique Match (%)**	**Multi-position Match (%)**	**Total Unmapped Reads (%)**
581	43554089	84.66	77.56	7.10	15.34
582	45687896	85.29	78.41	6.89	14.71
583	45733358	82.68	76.23	6.45	17.32
584	45452294	84.61	78.50	6.11	15.39
585	43555385	82.86	76.04	6.82	17.14
586	45428991	84.19	78.26	5.94	15.81
587	45564371	83.41	76.80	6.61	16.59
588	45692961	84.16	78.07	6.09	15.84
671	45552073	85.11	77.91	7.20	14.89
672	43546650	83.76	77.23	6.53	16.24
673	45731118	79.29	70.46	8.84	20.71
674	45732433	83.78	77.60	6.18	16.22
675	45732195	82.10	75.61	6.48	17.90
676	45706848	84.12	77.18	6.94	15.88
677	45715115	84.41	78.28	6.13	15.59
678	43552454	82.24	76.51	5.73	17.76
691	45572013	83.71	76.64	7.07	16.29
692	43551133	83.26	76.50	6.75	16.74
693	45684433	84.69	77.78	6.91	15.31
694	45729108	82.98	76.35	6.63	17.02
695	43552926	83.34	76.54	6.80	16.66
696	45595273	85.49	79.07	6.43	14.51
697	45729918	82.68	75.71	6.97	17.32
698	45546777	85.18	78.79	6.40	14.82
Total Mapped Reads (%)=Unique Match (%)+Multi-position Match (%).					
